# An enhanced recombinant amino‐terminal acetylation system and novel *in vivo* high‐throughput screen for molecules affecting α‐synuclein oligomerisation

**DOI:** 10.1002/1873-3468.12597

**Published:** 2017-03-06

**Authors:** Tara A. Eastwood, Karen Baker, Holly R. Brooker, Stefanie Frank, Daniel P. Mulvihill

**Affiliations:** ^1^ School of BiosciencesUniversity of KentCanterburyUK; ^2^ Present address: Departmental of Biochemical EngineeringUniversity College LondonLondonUK

**Keywords:** amino‐α‐acetyl‐transferase, Nat, Parkinson's disease, Tropomyosin, α‐synuclein

## Abstract

Amino‐terminal acetylation is a ubiquitous protein modification affecting the majority of eukaryote proteins to regulate stability and function. We describe an optimised recombinant expression system for rapid production of amino terminal‐acetylated proteins within bacteria. We go on to describe the system's use in a fluorescence based *in vivo* assay for use in the high‐throughput screen to identify drugs that impact amino‐terminal acetylation‐dependent oligomerisation. These new tools and protocols will allow researchers to enhance routine recombinant protein production and identify new molecules for use in research and clinical applications.

## **Abbreviations**

**BiFC**, Bimolecular Fluorescence Complementation

**IPTG**, isopropyl β‐d‐1 thiogalactopyranoside; Nt, Amino‐terminal

**Nat**, amino‐α‐acetyl‐transferase

**αSyn**, α‐synuclein

Amino‐terminal (Nt) acetylation is a ubiquitous co‐ and post‐translational modification affecting up to 90% of the eukaryote proteome 1. Eukaryote cells can possess up to six different amino‐α‐acetyl‐transferase complexes (Nat A‐F), each capable of conjugating an acetyl group onto the amino‐terminal residue of a protein, according to its amino‐terminal dipeptide 2. This global protein modification has diverse effects upon protein structure, stability and function by inhibiting amino proteolysis, modulating membrane interactions and protein folding and providing a signal for protein–protein interactions. These modifications impact a large number of cellular processes including cytoskeletal organisation, signalling, ER translocation, cell cycle progression, protein degradation and apoptosis 2, 3, 4, 5, as well as resistance to viral infection and disease states such as α‐synucleinopathies, heart disease and cancer 4, 6, 7, 8, 9. Thus, Nat complexes are attractive targets for drug‐based therapies.

α‐synuclein (αSyn) is an essential mammalian protein making up ~ 1% of the cytosolic proteome within neurons. It localises to presynaptic terminals and while its precise cellular function is unknown it is thought to somehow play a role in regulating vesicle turnover at synaptic termini 10. αSyn is largely unstructured when cytosolic, but in the presence of membranes it acquires an α‐helical folded structure capable of dimer and subsequent oligomer formation. This oligomerisation is in part modulated by an array of post‐translational modifications which include amino‐terminal acetylation and multiple phosphorylation events 11. Structural studies have indicated the stability of the amino‐terminal α‐helical structure and subsequent membrane interactions are regulated by Nt‐acetylation of the αSyn protein 8, 12, 13, 14, 15. These modifications, the oligomerisation status, and mutations within the *SNCA* gene (encodes for αSyn) are all major factors in αSyn fibril and aggregate formation, and each are key influences in the pathology of α‐synucleinopathies, such as Parkinson's disease.

Although Nt‐acetylation does occur in bacteria, it occurs to a significantly lesser extent when compared to eukaryotes 16. Prokaryotes lack the amino‐α‐acetyltransferase complexes required for efficient N‐terminal acetylation which occurs to the majority (~ 90%) of eukaryote proteins. In order to generate significant Nt‐acetylated proteins for subsequent biochemical and structural assays researchers rely upon either eukaryote expression systems or postpurification *in vitro* amino‐terminal acetylation reactions, each of which has significant time and cost implications. By applying a novel recombinant bacterial N‐terminal acetylation system developed within this lab 17 it is possible to acetylate the amino‐terminal residue of recombinant proteins, which has allowed researchers to establish this modification has a significant impact upon the structural conformation and binding properties of diverse proteins from a plethora of cell types 3, 6, 7, 8, 12, 13, 14, 15. This initial molecular tool for producing modified recombinant proteins was limited to its ability to only Nt‐acetylate substrates of the NatB complex (i.e. proteins starting with M‐D‐, M‐E‐, M‐N‐ or M‐Q‐). In addition the efficiency of substrate Nt‐acetylation when using this system was not 100% efficient for all target proteins, and therefore did not always permit the production and purification of a homogenous Nt‐acetylated substrate.

Here, we describe an expanded and improved recombinant Nt‐acetylation system where coexpression with either the fission yeast NatA or NatB complex allows the production of Nt‐acetylated proteins from *Escherichia coli* that allows modification of more than 50% of the eukaryote proteome with 100% efficiency. We go on to show this system has no detectable impact upon the health or expression levels of target proteins within the bacterial cell facilitating large‐scale production of Nt‐modified proteins. Furthermore, we describe how this screen can be applied in high‐throughput fluorescence‐based assays to identify drugs that impact protein oligomerisation by applying it to identify molecules which modulate αSyn amyloid formation.

## Materials and methods

### Extended optimised recombinant Nt‐acetylation system

Sequence optimised cDNA encoding for catalytic and regulatory subunits of the fission yeast NatA (*naa10* and *naa15*) and NatB (*naa20* and *naa25*) complexes were generated synthetically (Thermofisher, Waltham, MA USA) and cloned into pACYCDUET expression vectors (Novagen, Merck, Darmstadt, Germany) to generate pNatA and pNatB (available from http://www.addgene.org/Dan_Mulvihill/).

### Generation of Prha and BiFC expression vectors

The Prha promoter 18 was cloned into pET3a (Novagen) together with cDNA encoding for either the rabbit α‐skeletal muscle tropomosin (Tpm1.1) or mouse Tpm4.2 isoforms to create pRham‐αSkTm and pRham‐Tm4.2 respectively. cDNA encoding for the amino‐ and carboxyl‐half fluorophore fragments from an enhanced mVenus‐based BiFC system 19 were synthesised and cloned into the discrete multicloning sites of pETDUET‐1 (Novagen) to generate pET‐BiFC. αSyn cDNAs were amplified as *Nco1‐BamH1* and *Nde1‐BglII* fragments and ligated into pET‐BiFC to create pET‐αSyn‐BiFC.

### Induction of Nat complex and target substrate in *E. coli*

BL21(DE3) competent cells containing pNatA or pNatB (contain chloramphenicol selection markers) were generated and subsequently transformed with target protein expression plasmids. Induction cultures were inoculated from overnight starter cultures and allowed to grow with rapid shaking until reaching an OD_595_ of 0.4–0.5. For sequential induction, expression of the Nat complexes was induced by first culturing in 100 mg·L^−1^ IPTG for 2 h before adding L‐rhamnose (to a concentration of 0.2% w/v) to induce production of substrate protein. Glucose was added to the culture 1 h before cell harvesting to repress Prha‐dependent transcription and ensure all the synthesised target protein had associated with the Nat complex. During BiFC assays expression, Nat complex and BiFC tag‐labelled proteins were induced simultaneously by culturing in 20 mg·L^−1^ IPTG. Proteins were purified and subjected to electron‐spray mass spectroscopy to determine efficiency of amino‐terminal acetylation.

### Monitoring growth and fluorescence of cell cultures – plate readers and microscopy

Cell growth and fluorescence of cultures were followed in 96‐well fluorescence assay plates (Brand noncoated Puregrade S) using a BMG Spectrostar Nano plate reader, shaking cells at 37 °C. mCerulean3 and Venus BiFC fluorescent proteins were excited at 433 and 500 nm wavelengths respectively. Experimental cultures were inoculated from overnight starter cultures, which were grown to mid‐log phase and diluted in fresh media containing antibiotic and 20 mg·L^−1^ IPTG, to an OD_595_ of 0.01, and dispensed into the plate wells using an automated multidispense pipette. The outer most plate wells were not used and all readings were corrected for plate position‐dependent variation in fluorescence. Data points on all growth curves were calculated from averages of > 3 replicate cultures on the same plate. Each experiment was repeated on at least three independent occasions and representative curves are shown here.

## Results

### Nt‐acetylation system

**Figure 1:**
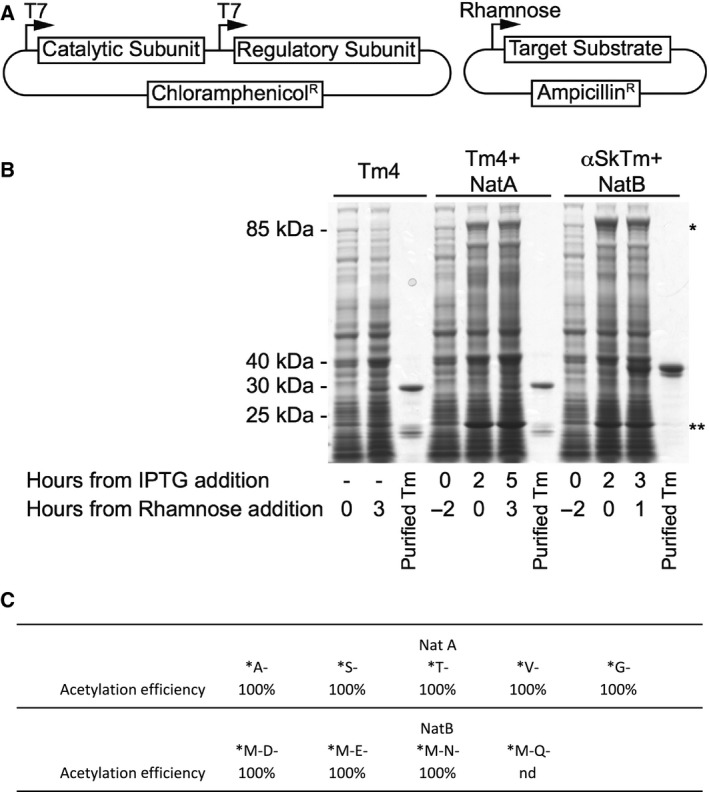
Bacterial Nt‐acetylation expression system. (A) Schematic outline of Nat constructs containing genes encoding catalytic and regulatory subunits of the each Nt‐acetylation complex under the control of T7 promoters. Expression of the substrate from a separate construct is under the control of the rhamnose promoter. (B) Cell lysates from BL21(DE3) cells containing either pRham‐Tm4.2 (left), pRham‐Tm4.2 and pNatA (middle), or pRham‐αSkTm and pNatB (right) were separated by SDS/PAGE following sequential addition of IPTG and Rhamnose, and visualised using coomassie stain. * and ** denote bands corresponding to Nat regulatory and catalytic subunits respectively. (C) Summary of amino‐termini tested and Nt‐acetylation efficiency. Culturing cells at lower temperatures as well as reducing the strength of target protein induction (by adding reduced quantities of IPTG/rhamnose) also resulted in improved Nt‐acetylation efficiency. We have observed 100% Nt‐acetylation of a number of target proteins (e.g. Tpm, Calmodulin) when cells are cultured in minimal media (e.g. for ^15^N labelling of proteins), however, isotope‐enriched rich media (NZY) can improve Nt‐acetylation in some cases.

We have previously described a recombinant Nt‐acetylation system for producing Nt‐modified proteins within the *E. coli* cell. Although this worked well for some NatB substrates, such as the fission yeast tropomyosin, its use often resulted in only a subpopulation of the purified target protein having the amino‐terminal methionine modified, sometimes as little as 25% 17. To improve the efficiency of the recombinant Nt‐acetylation system and extend the technology to enable modification of the majority of eukaryotic proteomes we generated new constructs. These expressed sequence optimised components of the fission yeast NatA and the NatB complexes (Fig. 1A) and in combination these complexes are responsible for the Nt‐acetylation of more than 50% of the eukaryotic proteome 2. The amino‐termini of NatA substrates (e.g. M‐A‐, M‐T‐, M‐S‐, etc.) are processed by a methionine aminopeptidase, present in *E. coli* 20, prior to Nt‐acetylation of the subsequent terminal residue. SDS/PAGE of cell extracts from *E. coli* cells containing the pNatA or pNatB constructs revealed strong and clearly defined bands that migrated at the expected sizes for each components of the Nat complexes (Fig. 1B), the majority of which was soluble (not shown).

As well as optimising the expression of the Nat proteins to improve the efficiency of the system, we decided to employ bacterial expression constructs in which transcription of the gene encoding for a recombinant protein can be induced independently of the Nat complexes. By putting the expression of the target protein under the control of the Prha rhamnose‐inducible promoter 21, not only are the Prha and T7 promoters triggered independently of each other, allowing sequential induction of different proteins within the cell, but postinduction transcription from the Prha promoter can be subsequently suppressed by the addition of glucose to the growth media. We made use of this to precisely control the sequential induction of the Nat complex followed by the transient production of the protein to be acetylated (Fig. 1B). This strategy ensures the Nat complex is present in excess during expression of its substrate and allows the researcher to repress induction of the target protein for a period before harvesting to ensure that all of the target Nat substrate proteins are Nt‐acetylated prior to cell harvesting.

### Validation of expanded Nt‐acetylation system

The efficiency of the new bacterial Nt‐acetylation system was tested upon different substrates of the NatA and NatB complex. Tropomyosins are conserved cytoskeletal proteins and Nt acetylation is critical for their proper structural organisation, localisation and function 22. Sequential induction of the appropriate optimised Nat complex followed by expression and repression of different Tropomyosins resulted in Nt‐acetylation of 100% of the purified isoforms tested. Similar efficiencies were also observed with proteins with representative amino‐terminal sequences for each Nat substrate, including calmodulin and the neuronal protein, α‐synuclein (Fig. 1C). The diversity of amino‐terminal sequences between these proteins illustrates the efficiency and versatility of this new recombinant modification system, and permits Nt‐acetylation of the majority of eukaryotic proteins within *E. coli*.

### The Nt‐acetylation systems do not impact cell growth or production of folded recombinant proteins

**Figure 2:**
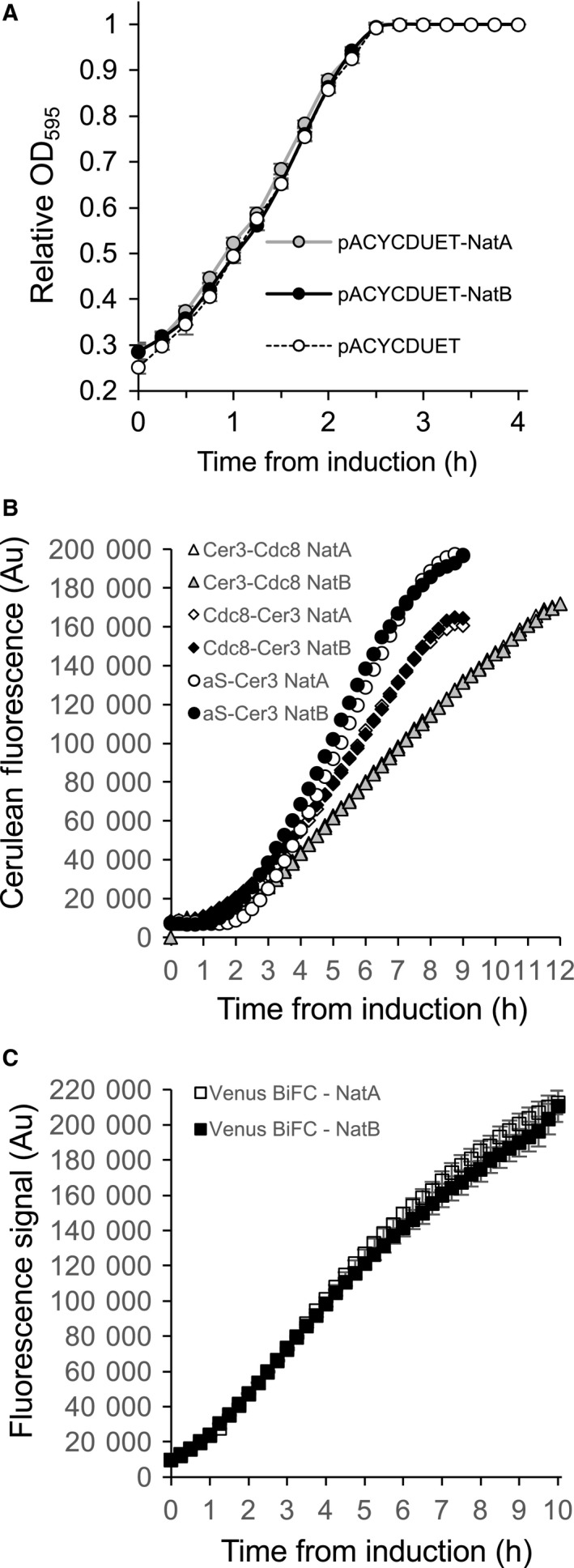
Bacterial Nt‐acetylation systems do not inhibit cell growth or production of target substrate proteins. (A) Growth curves generated from averages from 10 replicate cultures of BL21(DE3) *E. coli* cells containing an empty pACYCDUET vector (dashed lines and empty circles), pNatA (grey lines & grey filled circles) or pNatB (black lines and black filled circles), all grown on the same 96‐well plate. Expression of NatA or NatB does not impact bacterial growth. (B) Cerulean fluorescence signal from cultures of BL21(DE3) cells contained pJC20‐*cerulean3‐cdc8* (triangles), pJC20‐*cdc8‐cerulean3* (diamonds) pJC20‐α*Syn‐cerulean3* (circles) together with either pNatA (empty shape) or pNatB (filled shape). Expression of NatA or NatB does not impact expression of coexpressed recombinant proteins. (C) Yellow fluorescence signal from BL21(DE3) cells containing pET‐BiFC together with either pNatA (empty square) or pNatB (filled squares) illustrate Nt‐acetylation does not impact BiFC fluorescence.

Recombinant expression of Nat complexes has the potential to lead to a synthetic modification to the bacterial proteome, which could impact cell growth. However, growth curves generated from *E. coli* containing either pNatA, pNatB or the empty parental pACYCDUET vector were indistinguishable (Fig. 2A). Similarly, levels of correctly folded substrates which had been fused to fluorescent proteins (as measured by fluorescence – Fig. 2B) or cell growth (not shown) were unaffected by coexpression with the different Nat complexes. Not only were the expression of substrates tagged at their carboxyl terminus with the monomeric fluorescent protein, Cerulean3, unaffected (Cdc8‐Cer3/αSyn‐Cer3) but these data show that the NatA‐dependent Nt‐acetylation of the fluorescent protein when fused to the amino terminus of the fission yeast Tpm (Cer3‐Cdc8) does not affect protein expression, folding or fluorescence.

### A BiFC‐based method for monitoring Nt‐acetylation‐dependent interactions and oligomerisation

Bimolecular Fluorescence Complementation (BiFC) is a powerful tool for exploring protein–protein interactions within a cell. In this assay, cells express the two halves of a fluorescent protein fused to two potential interacting proteins. If these come into close proximity with each other in an appropriate conformation the fluorophore halves can also be brought together to form a stable functional fluorophore. Using this assay in combination with the recombinant Nt‐acetylation system provides an elegant and simple *in vivo* system to screen for modulators of Nt‐acetylation‐dependent complex formation within *E. coli*. To confirm the validity of any Nt‐acetylation‐dependent variation in BiFC signal a BiFC construct was generated encoding for each half of an enhanced Venus‐based BiFC reporter 19. No differences were observed in Venus fluorescence between cultures of *E. coli* cells expressing the BiFC fluorophore fragments and either Nat complex. This illustrates that NatA or NatB amino‐terminal acetylation does not affect the fluorescence or stability of the BiFC complex (Fig. 2C).

The amino‐terminal α‐helical domain of αSyn and subsequent oligomer formation is stabilised by Nt‐acetylation 13. Consistent with this αSyn oligomers are observed in boiled and SDS‐treated extracts from NatB complex containing bacterial cells, a significant proportion of which was observed in the pellet fraction after high speed centrifugation (not shown). Thus, NatB‐dependent Nt‐acetylation promotes stable αSyn oligomer formation within *E. coli*. A BiFC‐based assay therefore was developed to monitor this NatB‐dependent α‐synuclein oligermisation within *E. coli* cells.

**Figure 3:**
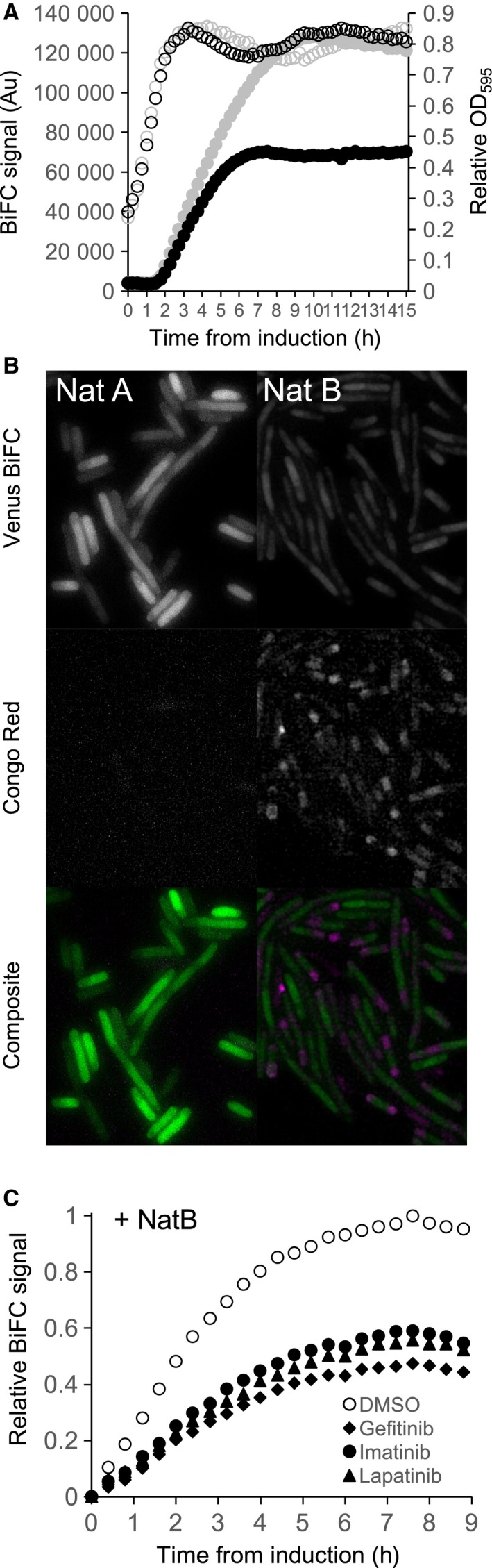
An *in vivo* BiFC system to identify Nt‐acetylation state‐specific αSyn oligomerisation. (A) Growth (empty circles) and fluorescence (filled circles) curves of BL21(DE3) cells containing pET‐αSyn‐BiFC together with either pNatA (grey shapes) or pNatB (black shapes) show while αSyn Nt‐acetylation does not impact cell growth (OD_595_), it affects BiFC signal from αSyn oligomers (Au). (B) αSyn‐BiFC fluorescence (upper panels, green bottom panels) and Congo Red staining (middle panels, magenta bottom panels) of BL21(DE3) cells containing pET‐αSyn‐BiFC with either pNatA (left panels) or pNatB (right panels) indicate Nt‐acetylation promotes αSyn amyloid formation in bacteria. (C) Fluorescence (filled circles) curves of BL21(DE3) cells containing pET‐αSyn‐BiFC and pNatB grown in the presence of DMSO (empty circles), 5 mm Gefitinib (filled diamonds), 5 mm Imatinib (filled circles) or 5 mm Lapatinib (filled triangles) illustrate this class of tyrosine kinase inhibitors promote formation of amyloids of Nt‐acetylated αSyn.

A BiFC construct was generated encoding for a pair of αSyn carboxyl terminal fusions conjugated to each half of the same enhanced Venus‐based BiFC reporter 19. Venus fluorescence from cultures of NatB cells containing the αSyn‐BiFC reporter plateaued within 4 h. Fluorescence from equivalent NatA cells increased to twice the signal within the same time frame (Fig. 3A). This dramatic difference in BiFC signal was not reflected in any measurable difference in fusion protein production (Fig. S1) or cell growth rate (Fig. 3A). This indicates NatB‐dependent acetylation of αSyn promotes an oligomeric conformation where the carboxyl regions of the αSyn carboxyl termini are in a distinct conformation to that of the nonacetylated state.

This Nt‐acetylation‐dependent difference in signal was also observed when the cells were visualised by live‐cell imaging. A strong αSyn‐BiFC signal was observed throughout the cytosol of cells lacking the NatB complex (Fig. 3B). This signal was significantly lower in NatB cells and was associated with the appearance of Congo Red (Fig. 3B) or Niad 4 (not shown) stained amyloid structures 23. This is consistent with recent structural studies which show that Nt‐acetylated αSyn forms stable antiparallel oligomers, which have been suggested initially interact together as tetramers 24 and subsequently form larger plaque‐like structures 15. However, the precise impact Nt‐acetylation has upon the αSyn structure and oligomerisation remains a matter of significant debate. In contrast, unmodified αSyn is more disordered and can align parallel to each other, resulting in an increased BiFC signal, and have a reduced propensity to incorporate into larger complex structures 25, 26 (Fig. S1).

### A high‐throughput screen for αSyn oligomerisation affecters

Molecules that modulate aggregate formation provide attractive potential therapeutic agents for treating α‐synucleinopathies 27, 28, 29. Having established this αSyn‐BiFC system provides a simple mechanism to monitor αSyn oligomer and plaque formation within the overnight cultures of bacterial cells, we decided to test whether it could be used to screen for molecules that impact this Nt‐acetylation‐dependent complex organisation. A preliminary screen was undertaken using a bank of FDA‐approved drugs available within the lab. While none of the drugs tested affected bacterial cell growth a subset of tyrosine kinase inhibitors specifically reduced the BiFC signal from Nt‐acetylated αSyn (Fig. 3C). In contrast, the same molecules did not affect the BiFC signal, and therefore conformation, of nonacetylated αSyn (not shown), also indicating they did not disrupt BiFC‐dependent fluorescence. This group of cancer therapy drugs have previously been shown to not only impact amyloid formation by inhibiting αSyn kinases (such as C‐Abl) and promote degradation *in vivo* but also improve motor neuron activity in α‐synucleinopathy models 30, 31, 32.

These data provide evidence to validate this simple and rapid high‐throughput screening method to identify molecules that change the rate of Nt‐acetylation‐dependent oligomer formation. This can be applied with automated high‐throughput technologies to identify novel therapies for Parkinson's disease and other Nat activity associated pathologies 9, 33.

## Discussion

Here, we describe a simple, quick, efficient and cheap method for producing amino‐terminally acetylated proteins from *E. coli*. We have improved the capability of an earlier system and provide the opportunity to Nt‐acetylate more than 50% of the proteome. In this method, the protein only has to be expressed and purified as normal from the cell, and does away with need for additional, often complex and inefficient *in vitro* acetylation steps. This system provides significant benefits over *in vitro* Nt‐modification systems in both time (these reactions are not 100% efficient, take significant time to optimise and require additional purification steps, where protein yield is reduced) and costs (while acetyl‐coA, the source of the acetyl group, is abundant within the *E. coli,* it is costly to purify or purchase).

We have shown that expression from the Nat complexes does not impact the health or growth rate of the bacterial culture, nor does it reduce the amount of target protein that is expressed within the *E. coli*. In addition, we and others have reported that Nt acetylation also stabilises protein in the bacterial cells and results in an increased yield 17, thus providing an opportunity to improve efficiency.

In this improved system we have also introduced a sequential induction system which allows the target recombinant protein to be induced when the Nat complex is in abundance within the *E. coli* cytosol. This is achieved through differential transcriptional control of the Nat complex and target proteins. In this way the target protein is induced after the Nat complex, and repressed before cell harvesting, while the Nat complex is still being produced.

Interestingly, as reported elsewhere, we have found that the use of minimal media and reduced culture temperatures (20 °C) can lead to improved quality and Nt‐acetylation efficiency of target proteins 12, as this is likely to slow down peptide translation and also facilitate improved protein folding.

We also describe a new Bi‐Molecular Fluorescence Complementation assay for monitoring Nt‐acetylation‐dependent oligomerisation of proteins. In this assay it was possible to monitor the oligomerisation state of the human neuronal protein, α‐synuclein, and formation of amyloid structures within the bacterial cell. The molecular‐genetic plasticity allowed by the bacterial cell allows the opportunity to simply determine which key factors (such as membrane lipid composition, temperature, stress, etc.) are critical in promoting the fibril or amyloid forms of α‐Syn. This BiFC Nat assay method is now being applied within this lab to study Nt‐acetylation regulation‐dependent oligomerisation of diverse proteins.

Finally, we go on to test and validate the use of this *in vivo* BiFC oligomerisation monitoring system in screens which can be used to not only identify inhibitors of Nt‐acetylation but also to identify molecules that impact the Nt‐acetylation‐dependent oligomer formation. In a blind trial screen we identified a series of related Tyrosine kinase inhibitors that promoted the formation of α‐Syn amyloids within the cell. No effect was seen on the unacetylated form, indicating the drugs did not impact protein production, cell viability or interaction between the BiFC fragments. A number of these drugs have been shown to affect amyloid formation within neuronal cell systems, as well as improve motor neuron activity in α‐synucleinopathy models, thus providing validation for this *E. coli* based drug screen. This provides a simple, cheap and rapid alternative to current screens (for example 28, 29) and when used together to validate findings provide a formidable tool‐kit to identify new and improved therapies against these debilitating diseases.

## Supporting information


**Fig. S1.** (A) Commassie stain (upper panel) and anti‐αSyn western blot (lower panel) analysis of extracts of BL21(DE3) pET‐αSyn‐BiFC cells expressing either NatA (left) or NatB (right). Samples were taken at 0, 1, 3, 5 and 7 h after IPTG addition. (B) Model of potential conformations of unmodified (upper figure) and Nt‐acetylated (lower figure) αSyn BiFC proteins.
