# Supplementary figures and images for: An enhanced recombinant amino‐terminal acetylation system and novel in vivo high‐throughput screen for molecules affecting α‐synuclein oligomerisation
Source: FEBS Lett. 2017 Mar 6;591(6):833–41. doi: 10.1002/1873-3468.12597 (PMC5396276; doi:10.1002/1873-3468.12597)

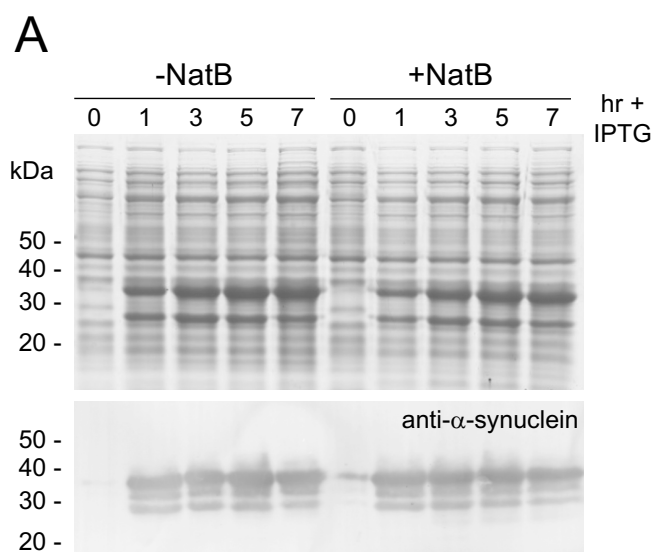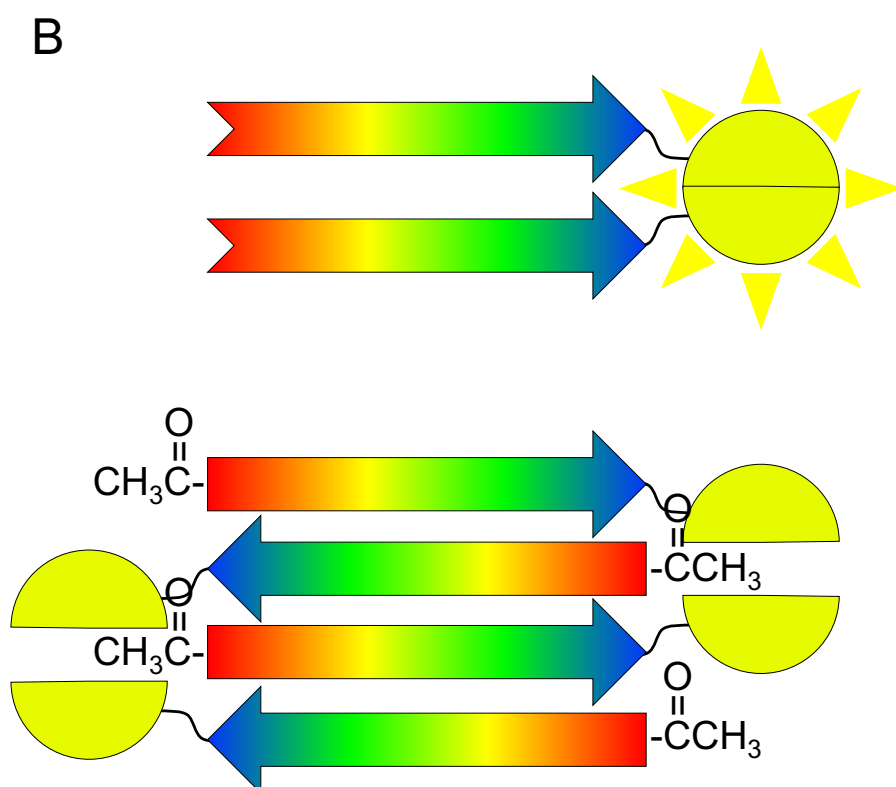

Figure S1

Supplement: Supplementary file 1 — Fig. S1. (A) Commassie stain (upper panel) and anti‐αSyn western blot (lower panel) analysis of extracts of BL21(DE3) pET‐αSyn‐BiFC cells expressing either NatA (left) or NatB (right). Samples were taken at 0, 1, 3, 5 and 7 h after IPTG addition. (B) Model of potential conformations of unmodified (upper figure) and Nt‐acetylated (lower figure) αSyn BiFC proteins. [file FEB2-591-833-s001.pdf]
